# Association between human herpesvirus 6 status and sarcopenia risk: a UK biobank cohort study with sex-specific patterns and telomere length modification

**DOI:** 10.3389/fimmu.2025.1623291

**Published:** 2025-09-17

**Authors:** Xiangliang Liu, Wang Yang, Xinqiao Chen, Yuting Liu, Yixin Zhao, Yuguang Li, Naifei Chen, Jiuwei Cui

**Affiliations:** Cancer Center, The First Hospital of Jilin University, Changchun, China

**Keywords:** human herpesvirus 6, sarcopenia, sex differences, telomere length, UK biobank

## Abstract

**Background:**

Sarcopenia represents a significant global health concern affecting older adults, yet its relationship with infectious agents remains poorly understood. This study investigated the association between human herpesvirus 6 (HHV-6) status and sarcopenia risk, examining potential sex-specific differences and biological modifiers.

**Methods:**

We analyzed data from 339,085 UK Biobank participants for baseline assessment and 27,030 participants for follow-up analysis. HHV-6 status was determined using TaqMan qPCR assay targeting conserved viral regions (DR1 and U7). Sarcopenia was defined according to European Working Group on Sarcopenia in Older People 2 (EWGSOP2) criteria. Multivariable logistic and Cox proportional hazards regression models were employed to assess associations, adjusting for comprehensive demographic, behavioral, and clinical covariates.

**Results:**

Individuals with DR-only positive HHV-6 status exhibited significantly elevated odds of sarcopenia at baseline (OR = 3.77, 95% CI: 1.44-8.08) and approximately fivefold increased risk during follow-up (HR = 4.76, 95% CI: 1.19-19.10). Sex-stratified analyses revealed pronounced male vulnerability to DR-only positivity (OR = 5.23, 95% CI: 1.74-12.60), while females showed associations only with typical positive status (OR = 1.63, 95% CI: 1.00-2.49). Telomere length significantly modified these relationships, with stronger associations among males with longer telomeres (OR = 6.57, 95% CI: 1.43-30.16) and females with shorter telomeres (OR = 1.94, 95% CI: 1.08-3.49). Results remained consistent across sensitivity analyses using alternative sarcopenia definitions.

**Conclusions:**

This study identifies novel associations between HHV-6 status, particularly DR-only positivity, and increased sarcopenia risk in a sex-specific manner. These associations are further modified by telomere length, indicating potential interactions between viral integration, cellular senescence, and muscle health. Our findings contribute to emerging research on infectious correlates of age-related muscle deterioration and may inform future investigations into preventive strategies.

## 1 Introduction

Sarcopenia, characterized by the progressive loss of skeletal muscle mass and function, represents a significant global health concern affecting approximately 10% of older adults worldwide (1, 2). Recognized as a geriatric syndrome with substantial clinical implications, sarcopenia contributes to increased risk of falls, functional decline, disability, poor quality of life, and premature mortality (3, 4). The European Working Group on Sarcopenia in Older People 2 (EWGSOP2) diagnostic algorithm defines sarcopenia through the concurrent presence of reduced muscle strength and diminished skeletal muscle mass, with severe sarcopenia further characterized by impaired physical performance (5). While age-related physiological changes remain the primary driver of sarcopenia development, growing evidence suggests that various modifiable and non-modifiable factors—including chronic inflammation, metabolic disorders, malnutrition, and genetic predisposition—significantly influence its pathogenesis (6, 7).

In recent years, increasing attention has focused on the potential role of infectious agents, particularly viruses with persistent or latent infection profiles, in age-related degenerative conditions (8, 9). Human herpesvirus 6 (HHV-6), a ubiquitous beta-herpesvirus with a high global seroprevalence exceeding 90% in adults, exhibits distinct biological characteristics that may have implications for chronic health conditions (10). HHV-6 exists in two closely related variants (HHV-6A and HHV-6B) and demonstrates unique genomic integration capabilities, with approximately 1% of the population carrying chromosomally integrated HHV-6 (ciHHV-6) (11, 12). This integration can manifest as different molecular phenotypes, including “typical positive” (with integration of both the direct repeat [DR] and unique [U] regions) and “DR-only positive” (with selective integration or amplification of DR sequences) (13, 14).

Chronic viral infections may contribute to sarcopenia pathophysiology through several biological mechanisms. Persistent viral presence can induce sustained low-grade inflammation, characterized by elevated pro-inflammatory cytokines (IL-6, TNF-α) that promote protein catabolism and impair muscle regeneration (15). Viral infections may also accelerate cellular senescence processes through oxidative stress pathways and telomere attrition, potentially connecting viral burden with accelerated biological aging (16, 17). HHV-6, specifically, has been associated with altered immune function, systemic inflammation, and tissue-specific pathologies that could theoretically influence muscle homeostasis (18, 19). Furthermore, emerging evidence suggests sex-specific differences in both viral immune responses and sarcopenia manifestation, highlighting the importance of gender-stratified analyses in investigating such associations (20, 21).

Despite these potential mechanistic links, the relationship between HHV-6 infection status and sarcopenia remains largely unexplored. Previous investigations have examined associations between cytomegalovirus (another herpesvirus) seropositivity and frailty or physical function (22), but specific studies addressing HHV-6 and skeletal muscle health are notably absent from the literature. Additionally, the potential modifying effects of important biological factors—such as systemic inflammation, body composition, genetic susceptibility, and telomere length—on virus-mediated muscle pathology have not been adequately characterized (23, 24).

Given these knowledge gaps, this study aimed to comprehensively investigate the association between HHV-6 status and sarcopenia using the UK Biobank cohort. Specifically, we sought to: 1) determine whether different HHV-6 integration profiles are associated with sarcopenia risk; 2) explore potential sex-specific differences in these associations; 3) examine the modifying effects of biological factors including inflammation markers, body mass index, genetic predisposition, and telomere length; and 4) establish temporal relationships through both cross-sectional and longitudinal analyses. By elucidating these relationships, this study contributes to the growing understanding of potential infectious determinants of sarcopenia and may inform future preventive and therapeutic strategies.

## 2 Methods

### 2.1 Study design and population

**Figure 1:**
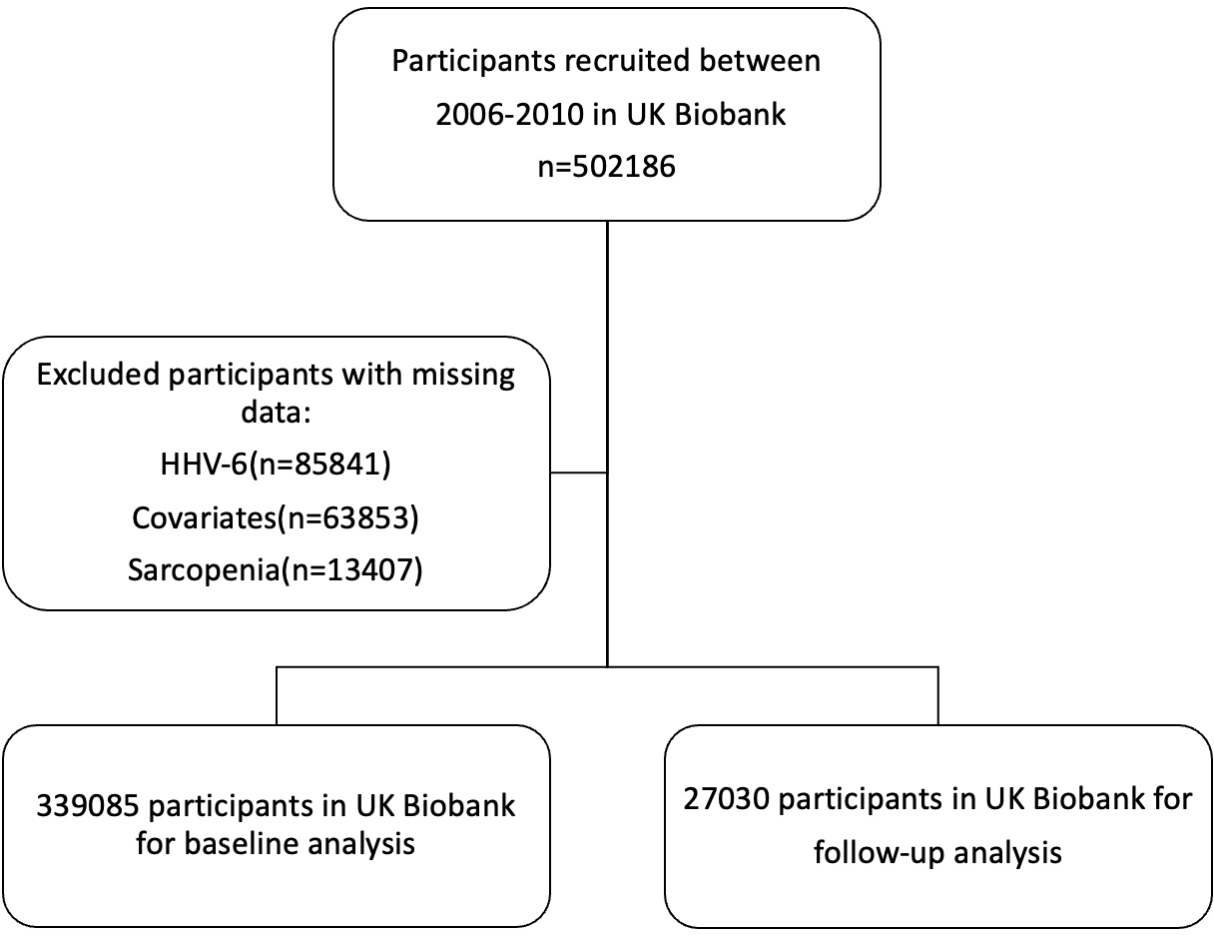
Study flowchart.

The UK Biobank is a large prospective study including more than half a million participants recruited between 2006 and 2010. During the enrollment phase, all participants engaged with a digital touch-screen interface to provide demographic information, lifestyle habits, and medical history, followed by standardized physical measurements and the collection of biological samples for analysis (25). Baseline demographic data, information on lifestyle, disease history, and physiological measurements were collected. After the exclusion of individuals with insufficient HHV-6 information (n=85841), missing data on covariates (n=63853), or insufficient information on sarcopenia at baseline (n=13407), the baseline analytic cohort comprised 339085 participants, while 27030 participants were included in the follow-up cycle deriving from UK Biobank’s imaging visit study (**Figure 1**).

### 2.2 HHV-6 assessment

DNA samples from over 416000 UK Biobank participants were screened using TaqMan qPCR assay targeting conserved regions of HHV-6 (DR1 and U7) alongside the single-copy human β-globin gene as an endogenous reference (26). The assay utilized hydrolysis probes for DR1 (FAM-labeled) and U7 (VIC-labeled), combined with β-globin (JUN-labeled), enabling simultaneous amplification and quantification. Positive controls (iciHHV-6A/B cell lines) and negative controls (iciHHV-6-negative DNA) were included in each reaction plate, with samples showing β-globin Ct >28 or evidence of contamination excluded. Classification of iciHHV-6 status was automated using ΔCt thresholds in accordance with UK Biobank TaqMan Assay protocols (β-globin Ct minus target Ct), with samples categorized as “Likely iciHHV-6” (Typical positive, ΔCt <6 for both DR1 and U7), “DR-only” (ΔCt DR1 <6/U7 ≥6), “U-only” (ΔCt U7 <6/DR1 ≥6), “Unlikely iciHHV-6” (6 ≤ ΔU7 Ct or 6 ≤ ΔDR1 Ct < 8) or “Negative” (ΔCt ≥8 for both). Incomplete or low-quality samples were removed in the data preprocessing procedure. The U-only subgroup was excluded from analyses as only one participant met this criteria.

### 2.3 Assessment of sarcopenia

In accordance with the European Working Group on Sarcopenia in Older People 2 (EWGSOP2) diagnostic algorithm (4), sarcopenia was defined by the concurrent presence of reduced muscle strength and diminished skeletal muscle mass. Muscle strength was quantified via baseline grip force measurements (averaged triplicate values from bilateral assessments) using a calibrated Jamar hydraulic hand dynamometer, with sex-specific thresholds set at <27 kg (males) and <16 kg (females) as per EWGSOP2 consensus criteria. Skeletal muscle mass was estimated through the skeletal muscle index (SMI), calculated as appendicular skeletal muscle mass (ASM) adjusted for stature (kg/height²). Low muscle mass was defined according to adapted EWGSOP2 criteria as <6.95 kg/m² for men and <5.30 kg/m² for women as previously described (27).

### 2.4 Covariates

Covariates comprised demographic (age, sex, ethnicity, education), behavioral (alcohol use, smoking status), biological (CRP [log mg/L], albumin [g/L]), and clinical factors (diabetes history, overall health rating). Furthermore, the standard Polygenic risk scores (PRS) set for BMI were derived using the UK Biobank’s PRS database, a framework aggregating cumulative genetic influences into an integrated metric of predisposition (28). Individuals were stratified into distinct genetic susceptibility categories according to PRS distribution percentile thresholds. The technically adjusted leucocyte telomere length(Z-adjusted T/S log) was collected from the UK Biobank, which is presented as a ratio of telomere repeat copy number to single copy gene (HBB) copy number, relative to a standard sample.

### 2.5 Statistical analysis

Baseline characteristics stratified by sarcopenia status were compared using Chi-squared tests for categorical variables and Student’s t-tests or Mann-Whitney U tests for continuous variables. Multivariable Logistic regression analysis quantified the relationship between baseline sarcopenia risk and HHV-6 status, reporting odds ratios (ORs) and 95% confidence intervals. Model 1 was the unadjusted crude model. Model 2 was further adjusted for demographics (age, sex, ethnicity). Model 3 was fully adjusted for smoking status, alcohol status, education level, history of diabetes, CRP level, albumin level, and overall health rating. In future sarcopenia risk analyses, Cox proportional hazards models were employed across analogous adjustment tiers using hazard ratios (HRs). Proportional hazards assumptions were verified via Schoenfeld residuals. Sex-stratified analyses tested gender-specific associations. Sensitivity analyses repeated models using grip strength as an alternative sarcopenia definition. Additional logistic regression analyses were performed to test associations between serostatus of Epstein-Barr virus(EBV) or cytomegalovirus(CMV) and sarcopenia. The restricted cubic spline (RCS) model was used to evaluate the nonlinear relationship between HHV-6 markers and sarcopenia. Subgroup analyses stratified participants by median CRP levels, BMI cutoffs (≥30 kg/m²), BMI PRS, and telomere length (Z-adjusted T/S ratio) to assess biological modifier effects. All analyses were performed in R version 4.2.1, and two-tailed p < 0.05 was defined as statistical significance.

## 3 Results

### 3.1 Baseline characteristics

339085 participants were included in the baseline cohort after excluding those with missing or invalid data, of which 2738 were identified as having sarcopenia. Participant characteristics are described in **Table 1**. Patients with sarcopenia tended to be older, male, low educated, Asian, HHV-6 positive, non-alcohol drinkers, diabetic, and had lower overall health rating. These individuals also had a lower level of albumin, a higher level of CRP. Additionally, 27030 participants were included in the follow-up cycle, of which 892 were identified as having sarcopenia (**Supplementary Table S1**). Similar characteristics was observed in participants diagnosed with sarcopenia in the follow-up cycle, with a significant propensity of HHV-6 DR positivity.

**Table 1:** Baseline characteristic.

Characteristic	Level	Non-sarcopenia (n=336347)	Sarcopenia (n=2738)	P
age (mean (SD))		56.543 (8.06)	61.978 (6.19)	<0.0001
Sex (%)	Female	181259 (53.89)	1062 (38.79)	<0.0001
	Male	155088 (46.11)	1676 (61.21)	
Ethnicity (%)	Asian	6685 (1.99)	325 (11.87)	<0.0001
	Black	2086 (0.62)	30 (1.10)	
	Mixed	1295 (0.39)	12 (0.44)	
	White	326281 (97.01)	2371 (86.60)	
Education level (%)	college	110604 (32.88)	405 (14.79)	<0.0001
	high school	169181 (50.30)	1136 (41.49)	
	less than high school	56562 (16.82)	1197 (43.72)	
TaqMan group (%)	Negative	331525 (98.57)	2688 (98.17)	0.0028
	DR-only positive	208 (0.06)	6 (0.22)	
	Typical positive	4614 (1.37)	44 (1.61)	
Alcohol status (%)	Current	311354 (92.57)	2211 (80.75)	<0.0001
	Never	13199 (3.92)	301 (10.99)	
	Prefer not to answer	264 (0.08)	9 (0.33)	
	Previous	11530 (3.43)	217 (7.93)	
Smoking status (%)	No	134253 (39.92)	1094 (39.96)	0.9807
	Yes	202094 (60.08)	1644 (60.04)	
Diabetes (%)	No	319999 (95.14)	2212 (80.79)	<0.0001
	Yes	16348 (4.86)	526 (19.21)	
Overall health rating (%)	Excellent	56890 (16.91)	107 (3.91)	<0.0001
	Fair	68503 (20.37)	1089 (39.77)	
	Good	197513 (58.72)	982 (35.87)	
	Poor	13441 (4.00)	560 (20.45)	
Albumin (mean (SD))		45.245 (2.61)	44.305 (2.83)	<0.0001
CRP (mean (SD))		2.552 (4.24)	5.214 (7.25)	<0.0001

### 3.2 Association between HHV-6 and sarcopenia

**Figure 2:**
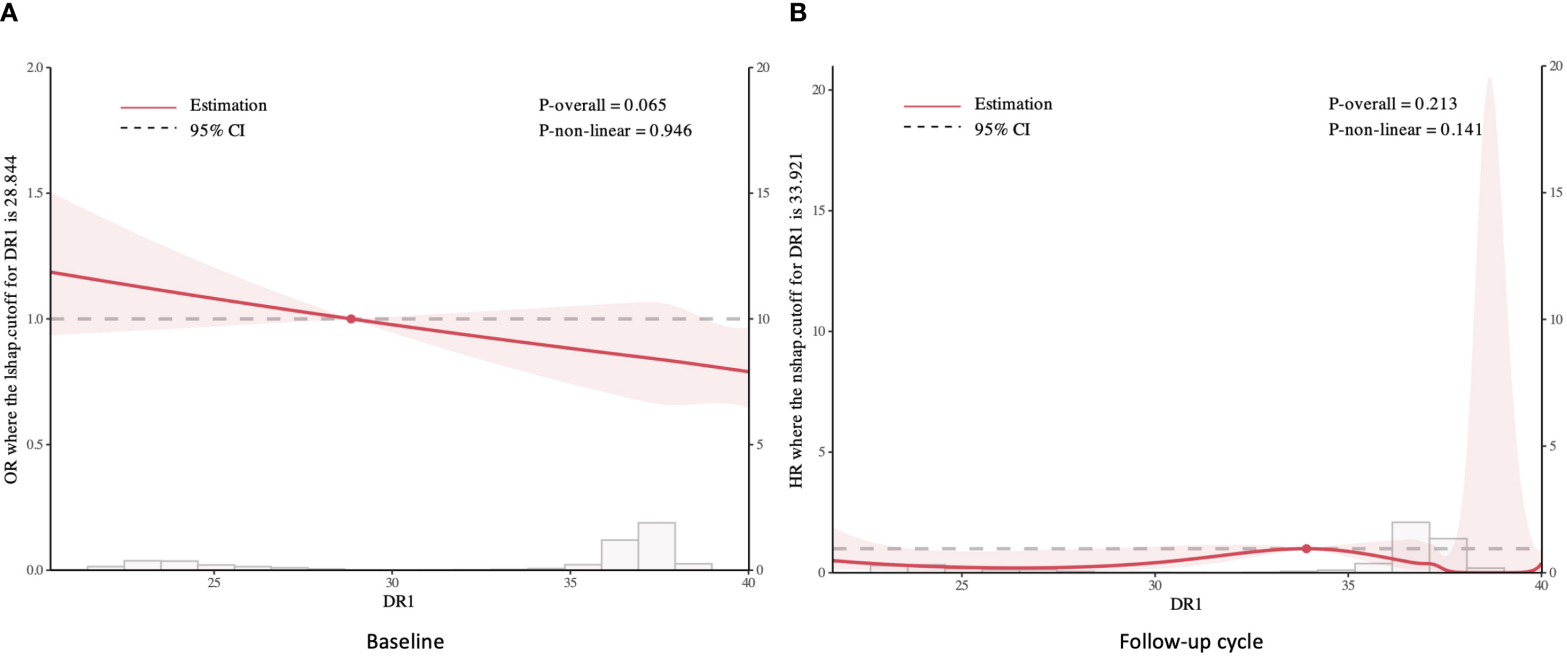
Restricted cubic spline analysis of sarcopenia risk by DR cycle threshold values: Baseline **(A)** and Follow-up cycle **(B)**.

The multivariable-adjusted Logistic regression modeling of covariate-adjusted associations between HHV-6 and baseline sarcopenia risks are shown in **Table 2**. Individuals with DR-only positive status exhibited significantly elevated odds of sarcopenia across all adjustment models, with ORs of 3.56(95% CI: 1.40,7.32), 3.90(95% CI: 1.53,8.12), and 3.77(95% CI: 1.44,8.08), respectively. In contrast, typical positive status showed no significant association with sarcopenia in all three models. Sex-stratified analyses revealed pronounced gender differences. Among males, DR-only positivity remained strongly associated with sarcopenia (OR = 5.68, 95% CI: 1.99–12.60; OR = 5.69, 95% CI: 1.98–13.00; OR = 5.23, 95% CI: 1.74–12.60; respectively), while females exhibited no significant association. Furthermore, typical positive status in females demonstrated a significant association after full adjustment (OR = 1.63, 95% CI: 1.00–2.49; p = 0.035), which was absent in males. Moreover, the restricted cubic spline analysis demonstrated a decreasing trend in sarcopenia risk with elevated cycle threshold (ct) value of DR, though the association remained statistically nonsignificant (**Figure 2A**).

**Table 2:** The Logistic regression analysis of HHV-6 status and baseline sarcopenia.

Characteristic	Model 1 (unadjusted)			Model 2 (demographics adjusted)			Model 3 (fully adjusted)		
	OR^1^	95% CI^1^	P-value	OR^1^	95% CI^1^	P-value	OR^1^	95% CI^1^	P-value
Baseline group									
Negative	—	—		—	—		—	—	
DR-only positive	3.56	1.40, 7.32	0.002	3.90	1.53, 8.12	0.001	3.77	1.44, 8.08	0.002
Typical positive	1.18	0.86, 1.57	0.3	1.29	0.94, 1.72	0.10	1.30	0.94, 1.74	0.094
Baseline female group									
Negative	—	—		—	—		—	—	
DR-only positive	1.38	0.08, 6.15	0.8	1.56	0.09, 7.03	0.7	1.53	0.09, 7.02	0.7
Typical positive	1.40	0.87, 2.12	0.14	1.52	0.94, 2.31	0.064	1.63	1.00, 2.49	0.035
Baseline male group									
Negative	—	—		—	—		—	—	
DR-only positive	5.68	1.99, 12.60	<0.001	5.69	1.98, 13.00	<0.001	5.23	1.74, 12.60	<0.001
Typical positive	1.04	0.67, 1.52	0.8	1.15	0.74, 1.68	0.5	1.12	0.72, 1.65	0.6

^1^OR, Odds Ratio; CI, Confidence Interval.

In the follow-up cohort, DR-only positivity showed a non-significant trend toward elevated risk in unadjusted models (HR = 3.75, 95% CI: 0.94–15.00; p = 0.062), which became significant after full adjustment (HR = 4.76, 95% CI: 1.19–19.10; p = 0.028, **Supplementary Table S2**). Similarly, sex-stratified analyses revealed robust associations exclusively in males. Fully adjusted models for males demonstrated a 7.24-fold increased risk (95% CI: 1.80–29.10; p = 0.005) among DR-only positive individuals, whereas no association was observed in females. In contrast, typical positive status showed no significant association with incident sarcopenia in any subgroup (all p > 0.05). Additionally, the restricted cubic splines revealed no significant association between the cycle threshold value of DR and sarcopenia in the follow-up group (**Figure 2B**).

### 3.3 Sensitivity and subgroup analyses

As shown in **Supplementary Figure S1**, a significant interaction was found between telomere length (Z-adjusted T/S ratio, low [L] vs. high [H] stratified by median) and HHV-6 status, and was strongly modified by sex. Among males with shorter telomeres, DR-only positivity yielded a 4.64-fold increased sarcopenia risk (OR = 4.64, 95% CI: 1.32–16.27; p = 0.017), whereas those with longer telomeres also showed a significant increase in sarcopenia risk, with an odds ratio of 6.57 (OR = 6.57, 95% CI: 1.43–30.16; p = 0.015). In females, no significant interaction was observed, with typical positive status associated only in shorter telomere subgroups (OR = 1.94, 95% CI: 1.08–3.49; p = 0.027). Conversely, subgroup analyses stratified by CRP and BMI demonstrated no effect modification. In higher CRP and BMI groups, DR-only positive males and typical positive females retained heightened sarcopenia risk.

Sensitivity analyses using grip strength as an alternative sarcopenia definition yielded consistent patterns, with DR-only positive males retained elevated risk at baseline and in the follow-up cycle (OR = 2.71, 95% CI: 1.23–5.28, p = 0.007; HR = 6.25, 95% CI: 2.00–19.50, p = 0.002; **Table 3**, **Supplementary Table S3**).

**Table 3:** Sensitivity analysis of the correlation between HHV-6 status and baseline sarcopenia.

Characteristic	Model 1 (unadjusted)			Model 2 (demographics adjusted)			Model 3 (fully adjusted)		
	OR^1^	95% CI^1^	P-value	OR^1^	95% CI^1^	P-value	OR^1^	95% CI^1^	P-value
Baseline group									
Negative	—	—		—	—		—	—	
DR-only positive	1.66	0.97, 2.64	0.045	1.70	0.99, 2.72	0.038	1.66	0.96, 2.69	0.052
Typical positive	1.06	0.92, 1.20	0.4	1.10	0.96, 1.25	0.14	1.12	0.98, 1.27	0.10
Baseline female group									
Negative	—	—		—	—		—	—	
DR-only positive	1.13	0.51, 2.16	0.7	1.19	0.53, 2.30	0.6	1.14	0.50, 2.22	0.7
Typical positive	1.03	0.86, 1.21	0.8	1.06	0.89, 1.25	0.5	1.09	0.91, 1.29	0.3
Baseline male group									
Negative	—	—		—	—		—	—	
DR-only positive	2.67	1.25, 5.04	0.005	2.71	1.26, 5.16	0.005	2.71	1.23, 5.28	0.007
Typical positive	1.10	0.90, 1.35	0.3	1.17	0.95, 1.43	0.12	1.16	0.93, 1.42	0.2

^1^OR, Odds Ratio; CI, Confidence Interval.

However, no statistically significant associations were observed between serostatus of EBV/CMV and sarcopenia, with wide confidence intervals reflecting limited statistical precision due to small seropositive subgroup sample sizes (**Supplementary Table S4**).

## 4 Discussion

This study provides novel evidence of a significant association between HHV-6 status and sarcopenia risk, with distinctive patterns observed across different viral integration profiles and between sexes. Our findings demonstrate that individuals with DR-only positive HHV-6 status exhibited nearly fourfold higher odds of sarcopenia at baseline and approximately fivefold elevated risk during follow-up after comprehensive adjustment for confounding factors. Notably, these associations were predominantly driven by pronounced effects in males, where DR-only positivity conferred over fivefold increased sarcopenia risk, while females showed significant associations only with typical positive status. Furthermore, telomere length significantly modified the HHV-6-sarcopenia relationship, with distinct interaction patterns observed between sexes.

The observed association between HHV-6 DR-only positive status and sarcopenia represents a novel finding in the field of muscle health. Previous investigations have primarily focused on CMV in relation to physical function and frailty (29, 30), with limited attention to other herpesviruses. Wang et al. demonstrated that CMV seropositivity was associated with increased frailty risk in older people (22), while Matheï et al. reported associations between CMV infection and inflammatory markers linked to physical decline (31). Our results extend this emerging field by identifying HHV-6, particularly the DR-only phenotype, as a potential viral determinant of muscle health. The selective association with DR-only positivity, rather than typical positive status, suggests that specific molecular mechanisms related to partial viral genome integration may contribute to muscle pathology.

The marked sexual dimorphism observed in our analyses aligns with growing evidence of sex-specific immunological responses to viral infections (32, 33). The pronounced association between DR-only positivity and sarcopenia in males may reflect sex-based differences in immune responses to latent viral infections. Testosterone has been shown to modulate pro-inflammatory cytokine production (34), potentially exacerbating muscle catabolism in the presence of persistent viral triggers. Conversely, the female-specific association with typical positive status suggests distinct pathophysiological pathways. Estrogen’s immunomodulatory effects may alter viral reactivation patterns (35), triggering different inflammatory cascades associated with complete viral integration. These findings underscore the importance of sex-stratified analyses in investigations of infectious determinants of age-related conditions.

Particularly intriguing was the interaction between telomere length and HHV-6 status in modifying sarcopenia risk. In males, DR-only positivity conferred elevated sarcopenia risk regardless of telomere length, though the effect was slightly more pronounced among those with longer telomeres. This counterintuitive finding challenges the traditional view that shorter telomeres, typically associated with cellular senescence and aging (36), would exacerbate pathological processes. One potential explanation involves the relationship between telomere biology and viral integration. HHV-6 demonstrates a unique tropism for telomeric regions (37, 38), potentially disrupting telomere function even when length appears preserved. Michael L Wood et al. found that HHV-6 integration can lead to telomere lengthening at DRL-T2 (39). The HHV-6 genome can be excised and reactivated in this manner. Therefore, the DR-only group may represent those with reactivation. Furthermore, viral integration into telomeric regions may impair the recruitment of telomere-associated proteins necessary for proper telomere maintenance (40), creating functional deficits independent of measured length. However, measured telomere length reflects only one aspect of telomere biology, and the association reported here is observational and does not establish causation. These observed interactions may reflect an underlying confounding factor or biological synergy that has yet to be identified.

The biological mechanisms underlying the observed associations likely involve complex interactions between viral persistence, inflammation, and muscle homeostasis. DR-only positivity, representing selective integration or amplification of direct repeat viral sequences, may disrupt host genomic stability through insertional mutagenesis (41, 42). Such genomic alterations could potentially affect genes involved in myogenesis or muscle protein synthesis. Additionally, viral integration may trigger chronic low-grade inflammation—a well-established contributor to sarcopenia pathogenesis (43). HHV-6 has been shown to induce pro-inflammatory cytokines including IL-6 and TNF-α (44), which promote protein catabolism and impair muscle regeneration. The sex-specific patterns observed may reflect differential inflammatory responses, with males typically exhibiting more pronounced pro-inflammatory profiles following immune challenges (29, 45).

Mitochondrial dysfunction represents another potential mechanistic link between HHV-6 and sarcopenia. HHV-6 proteins have been shown to localize within mitochondria, altering respiratory function and promoting oxidative stress (46, 47). Mitochondrial impairment is increasingly recognized as a central feature of sarcopenia (48), potentially explaining how viral integration contributes to muscle deterioration. Furthermore, cellular senescence, a biological process characterized by irreversible cell cycle arrest and pro-inflammatory secretory phenotype, may be accelerated by chronic viral presence (49), contributing to impaired satellite cell function and compromised muscle regenerative capacity.

While no significant associations were found between EBV/CMV serostatus and sarcopenia in our cohort (both p>0.05), these results must be interpreted with caution. Small subgroup sizes (n=33) led to extremely wide confidence intervals, severely limiting statistical power to detect modest effects. Consequently, we cannot conclusively rule out confounding or effect modification by these viruses. Previous studies suggest immunosenescence may be associated with reduced muscle mass and strength, but direct evidence establishing its causal relationship with sarcopenia remains limited and requires further investigation (50).

Our study has several strengths, including its large sample size, prospective design, and comprehensive assessment of potential confounding and modifying factors. The use of standardized sarcopenia definitions following EWGSOP2 criteria enhances the clinical relevance of our findings. Additionally, the application of sensitive detection methods for HHV-6 status allowed for differentiation between integration profiles, revealing associations that might have been missed in studies examining only viral serostatus. The consistent results across sensitivity analyses using alternative sarcopenia definitions further supports the robustness of our findings.

However, several limitations warrant consideration. First, despite adjustment for numerous covariates, residual confounding cannot be entirely excluded. Second, the predominantly Caucasian (>95%) UK Biobank population limits generalizability to other ethnic groups. While valuable for investigating within-population effects, results derived from such a homogeneous cohort cannot be assumed to translate directly to populations of different ancestral backgrounds or diverse ethnic groups. Future large-scale, intentionally diverse cohorts are needed to validate or refute these findings across globally representative populations. Third, while our follow-up analyses support temporal relationships, the observational nature of the study precludes definitive causal inferences. Fourth, our classification of HHV-6 status relied on predetermined ΔCt thresholds established by the UK Biobank; however, these thresholds lack independent validation in external cohorts. Furthermore, the absence of a universal gold-standard diagnostic criterion for iciHHV-6 detection may introduce potential misclassification bias. Finally, the relatively small number of DR-only positive cases, particularly in sex-stratified analyses, resulted in wide confidence intervals despite statistical significance. The observed effect size must therefore be interpreted with caution, as it may represent an exaggeration of the true association. Future studies with larger, adequately powered DR-only cohorts are essential to provide more reliable estimates.

The clinical implications of our findings are substantial. Identification of HHV-6 status, particularly DR-only positivity in males, may enhance risk stratification for sarcopenia development. This could facilitate targeted preventive interventions in high-risk individuals, potentially including anti-inflammatory approaches or specific exercise regimens. From a therapeutic perspective, our results raise the possibility that antiviral strategies might benefit select sarcopenia patients with evidence of HHV-6 integration, though this requires formal investigation. Furthermore, the interaction with telomere biology suggests potential value in combined approaches addressing both viral burden and cellular senescence processes.

Future research directions should include mechanistic studies investigating HHV-6 effects on myocyte function, satellite cell activity, and muscle regeneration. Larger prospective cohorts with repeated measures of both HHV-6 status and muscle parameters would enhance understanding of temporal relationships. Studies incorporating viral reactivation markers alongside integration status would provide insights into active versus latent infection effects. Additionally, interventional trials targeting inflammation in HHV-6 positive individuals at risk for sarcopenia could evaluate potential preventive approaches.

## 5 Conclusion

In conclusion, this study provides novel evidence linking HHV-6 status, particularly DR-only positivity, with increased sarcopenia risk in a sex-specific manner, with males showing pronounced vulnerability. These associations are further modified by telomere length, suggesting complex interactions between viral integration, cellular senescence, and muscle health. Our findings contribute to the emerging understanding of infectious determinants of age-related muscle deterioration and may inform future preventive and therapeutic strategies for sarcopenia.

## Data Availability

Publicly available datasets were analyzed in this study. This data can be found here: https://biobank.ndph.ox.ac.uk/showcase/index.cgi.

## References

[1] Zheng Y Feng J Yu Y Ling M Wang X. Advances in sarcopenia: mechanisms, therapeutic targets, and intervention strategies. Arch Pharm Res. (2024) 47:301–24. doi: 10.1007/s12272-024-01493-2, PMID: 38592582

[2] Cruz-Jentoft AJ Sayer AA. Sarcopenia. Lancet. (2019) 393:2636–46. doi: 10.1016/S0140-6736(19)31138-9, PMID: 31171417

[3] Yeung SSY Reijnierse EM Pham VK Trappenburg MC Lim WK Meskers CGM. Sarcopenia and its association with falls and fractures in older adults: A systematic review and meta-analysis. J Cachexia Sarcopenia Muscle. (2019) 10:485–500. doi: 10.1002/jcsm.12411, PMID: 30993881 PMC6596401

[4] Cruz-Jentoft AJ Bahat G Bauer J Boirie Y Bruyere O Cederholm T. Sarcopenia: revised European consensus on definition and diagnosis. Age Ageing. (2019) 48:16–31. doi: 10.1093/ageing/afy169, PMID: 30312372 PMC6322506

[5] Stuck AK Basile G Freystaetter G de Godoi Rezende Costa Molino C Lang W Bischoff-Ferrari HA. Predictive validity of current sarcopenia definitions (EWGSOP2, SDOC, and AWGS2) for clinical outcomes: A scoping review. J Cachexia Sarcopenia Muscle. (2023) 14:71–83. doi: 10.1002/jcsm.13161, PMID: 36564353 PMC9891988

[6] Li Y Liu C Shi J Zheng X Chen Y Liu X. The association of metabolic disorders and prognosis in cancer patients. BMC Cancer. (2025) 25:278. doi: 10.1186/s12885-025-13707-x, PMID: 39962450 PMC11834268

[7] Penna F Costamagna D Pin F Camperi A Fanzani A Chiarpotto EM. Autophagic degradation contributes to muscle wasting in cancer cachexia. Am J Pathol. (2013) 182:1367–78. doi: 10.1016/j.ajpath.2012.12.023, PMID: 23395093

[8] Franceschi C Salvioli S Garagnani P de Eguileor M Monti D Capri M. Immunobiography and the heterogeneity of immune responses in the elderly: A focus on inflammaging and trained immunity. Front Immunol. (2017) 8:982. doi: 10.3389/fimmu.2017.00982, PMID: 28861086 PMC5559470

[9] Pawelec G. Age and immunity: what is “immunosenescence”? Exp Gerontol. (2018) 105:4–9. doi: 10.1016/j.exger.2017.10.024, PMID: 29111233

[10] Berzero G Campanini G Vegezzi E Paoletti M Pichiecchio A Simoncelli AM. Human herpesvirus 6 encephalitis in immunocompetent and immunocompromised hosts. Neurol Neuroimmunol Neuroinflamm. (2021) 8(2):e942. doi: 10.1212/NXI.0000000000000942, PMID: 33587722 PMC7963435

[11] Pantry SN Medveczky PG. Latency, integration, and reactivation of human herpesvirus-6. Viruses. (2017) 9(7):194. doi: 10.3390/v9070194, PMID: 28737715 PMC5537686

[12] Tweedy J Spyrou MA Pearson M Lassner D Kuhl U Gompels UA. Complete genome sequence of germline chromosomally integrated human herpesvirus 6A and analyses integration sites define a new human endogenous virus with potential to reactivate as an emerging infection. Viruses. (2016) 8(1):19. doi: 10.3390/v8010019, PMID: 26784220 PMC4728579

[13] Kojima S Kamada AJ Parrish NF. Virus-derived variation in diverse human genomes. PloS Genet. (2021) 17:e1009324. doi: 10.1371/journal.pgen.1009324, PMID: 33901175 PMC8101998

[14] Kusakin AV Goleva OV Danilov LG Krylov AV Tsay VV Kalinin RS. The telomeric repeats of HHV-6A do not determine the chromosome into which the virus is integrated. Genes (Basel). (2023) 14(2):521. doi: 10.3390/genes14020521, PMID: 36833448 PMC9957103

[15] Keyvani H Zahednasab H Aljanabi HAA Asadi M Mirzaei R Esghaei M. The role of human herpesvirus-6 and inflammatory markers in the pathogenesis of multiple sclerosis. J Neuroimmunol. (2020) 346:577313. doi: 10.1016/j.jneuroim.2020.577313, PMID: 32673896

[16] Wight DJ Aimola G Beythien G Flamand L Kaufer BB. Impact of host telomere length on HHV-6 integration. Viruses. (2022) 14(9):1864. doi: 10.3390/v14091864, PMID: 36146670 PMC9505050

[17] Iida T Ito Y Kanazashi M Murayama S Miyake T Yoshimaru Y. Effects of Psychological and Physical Stress on Oxidative Stress, Serotonin, and Fatigue in Young Females Induced by Objective Structured Clinical Examination: Pilot Study of u-8-OHdG, u-5HT, and s-HHV-6. Int J Tryptophan Res. (2021) 14:11786469211048443. doi: 10.1177/11786469211048443, PMID: 34658624 PMC8512239

[18] Aimola G Beythien G Aswad A Kaufer BB. Current understanding of human herpesvirus 6 (HHV-6) chromosomal integration. Antiviral Res. (2020) 176:104720. doi: 10.1016/j.antiviral.2020.104720, PMID: 32044155

[19] Caselli E D’Accolti M Caccuri F Soffritti I Gentili V Bortolotti D. The U94 gene of human herpesvirus 6: A narrative review of its role and potential functions. Cells. (2020) 9(12):2608. doi: 10.3390/cells9122608, PMID: 33291793 PMC7762089

[20] Cortes CJ De Miguel Z. Precision exercise medicine: sex specific differences in immune and CNS responses to physical activity. Brain Plast. (2022) 8:65–77. doi: 10.3233/BPL-220139, PMID: 36448044 PMC9661359

[21] Jia S Zhao W Hu F Zhao Y Ge M Xia X. Sex differences in the association of physical activity levels and vitamin D with obesity, sarcopenia, and sarcopenic obesity: a cross-sectional study. BMC Geriatr. (2022) 22:898. doi: 10.1186/s12877-022-03577-4, PMID: 36434519 PMC9701059

[22] Wang GC Kao WH Murakami P Xue QL Chiou RB Detrick B. Cytomegalovirus infection and the risk of mortality and frailty in older women: a prospective observational cohort study. Am J Epidemiol. (2010) 171:1144–52. doi: 10.1093/aje/kwq062, PMID: 20400465 PMC2877470

[23] Codd V Denniff M Swinfield C Warner SC Papakonstantinou M Sheth S. Measurement and initial characterization of leukocyte telomere length in 474,074 participants in UK Biobank. Nat Aging. (2022) 2:170–9. doi: 10.1038/s43587-021-00166-9, PMID: 37117760

[24] Cohen S Nathan JA Goldberg AL. Muscle wasting in disease: molecular mechanisms and promising therapies. Nat Rev Drug Discov. (2015) 14:58–74. doi: 10.1038/nrd4467, PMID: 25549588

[25] Sudlow C Gallacher J Allen N Beral V Burton P Danesh J. UK biobank: an open access resource for identifying the causes of a wide range of complex diseases of middle and old age. PloS Med. (2015) 12:e1001779. doi: 10.1371/journal.pmed.1001779, PMID: 25826379 PMC4380465

[26] Available online at: https://biobank.ndph.ox.ac.uk/showcase/refer.cgi?id=2007 (Accessed September 9, 2025).

[27] Janssen I Heymsfield SB Baumgartner RN Ross R. Estimation of skeletal muscle mass by bioelectrical impedance analysis. J Appl Physiol (1985). (2000) 89:465–71. doi: 10.1152/jappl.2000.89.2.465, PMID: 10926627

[28] Available online at: https://biobank.ndph.ox.ac.uk/showcase/refer.cgi?id=1227 (Accessed September 9, 2025).

[29] Leng SX Margolick JB. Aging, sex, inflammation, frailty, and CMV and HIV infections. Cell Immunol. (2020) 348:104024. doi: 10.1016/j.cellimm.2019.104024, PMID: 31843200 PMC7002257

[30] Samson LD van den Berg SP Engelfriet P Boots AM Hendriks M de Rond LG. Limited effect of duration of CMV infection on adaptive immunity and frailty: insights from a 27-year-long longitudinal study. Clin Transl Immunol. (2020) 9:e1193. doi: 10.1002/cti2.1193, PMID: 33133599 PMC7586993

[31] Mathei C Vaes B Wallemacq P Degryse J. Associations between cytomegalovirus infection and functional impairment and frailty in the BELFRAIL Cohort. J Am Geriatr Soc. (2011) 59:2201–8. doi: 10.1111/j.1532-5415.2011.03719.x, PMID: 22092044

[32] Takahashi T Iwasaki A. Sex differences in immune responses. Science. (2021) 371:347–8. doi: 10.1126/science.abe7199, PMID: 33479140

[33] Ghosh S Klein RS. Sex drives dimorphic immune responses to viral infections. J Immunol. (2017) 198:1782–90. doi: 10.4049/jimmunol.1601166, PMID: 28223406 PMC5325721

[34] Maggio M Basaria S Ceda GP Ble A Ling SM Bandinelli S. The relationship between testosterone and molecular markers of inflammation in older men. J Endocrinol Invest. (2005) 28:116–9., PMID: 16760639

[35] Straub RH. The complex role of estrogens in inflammation. Endocr Rev. (2007) 28:521–74. doi: 10.1210/er.2007-0001, PMID: 17640948

[36] Daniali L Benetos A Susser E Kark JD Labat C Kimura M. Telomeres shorten at equivalent rates in somatic tissues of adults. Nat Commun. (2013) 4:1597. doi: 10.1038/ncomms2602, PMID: 23511462 PMC3615479

[37] Arbuckle JH Medveczky MM Luka J Hadley SH Luegmayr A Ablashi D. The latent human herpesvirus-6A genome specifically integrates in telomeres of human chromosomes *in vivo* and *in vitro* . Proc Natl Acad Sci U.S.A. (2010) 107:5563–8. doi: 10.1073/pnas.0913586107, PMID: 20212114 PMC2851814

[38] Flamand L. Chromosomal integration by human herpesviruses 6A and 6B. Adv Exp Med Biol. (2018) 1045:209–26. doi: 10.1007/978-981-10-7230-7_10, PMID: 29896669

[39] Wood ML Veal CD Neumann R Suarez NM Nichols J Parker AJ. Variation in human herpesvirus 6B telomeric integration, excision, and transmission between tissues and individuals. Elife. (2021) 10:e70452. doi: 10.7554/eLife.70452.sa2, PMID: 34545807 PMC8492063

[40] Wood ML Royle NJ. Chromosomally integrated human herpesvirus 6: models of viral genome release from the telomere and impacts on human health. Viruses. (2017) 9(7):184. doi: 10.3390/v9070184, PMID: 28704957 PMC5537676

[41] Gravel A Dubuc I Morissette G Sedlak RH Jerome KR Flamand L. Inherited chromosomally integrated human herpesvirus 6 as a predisposing risk factor for the development of angina pectoris. Proc Natl Acad Sci U.S.A. (2015) 112:8058–63. doi: 10.1073/pnas.1502741112, PMID: 26080419 PMC4491735

[42] Kaufer BB Flamand L. Chromosomally integrated HHV-6: impact on virus, cell and organismal biology. Curr Opin Virol. (2014) 9:111–8. doi: 10.1016/j.coviro.2014.09.010, PMID: 25462442

[43] Zhang J Wang Y Liu H Lei Z Cheng S Cao H. The association between eight complete blood count-derived inflammatory markers and muscle health. Front Nutr. (2025) 12:1498757. doi: 10.3389/fnut.2025.1498757, PMID: 39963665 PMC11830586

[44] Lu Y Liu BP Tan CT Pan F Larbi A Ng TP. Lifetime pathogen burden, inflammatory markers, and depression in community-dwelling older adults. Brain Behav Immun. (2022) 102:124–34. doi: 10.1016/j.bbi.2022.02.020, PMID: 35202734

[45] Hoffmann JP Liu JA Seddu K Klein SL. Sex hormone signaling and regulation of immune function. Immunity. (2023) 56:2472–91. doi: 10.1016/j.immuni.2023.10.008, PMID: 37967530

[46] Li L Chi J Zhou F Guo D Wang F Liu G. Human herpesvirus 6A induces apoptosis of HSB-2 cells via a mitochondrion-related caspase pathway. J BioMed Res. (2010) 24:444–51. doi: 10.1016/S1674-8301(10)60059-0, PMID: 23554661 PMC3596692

[47] Soffritti I D’Accolti M Bini F Mazziga E Di Luca D Maccari C. Virus-induced microRNA modulation and systemic sclerosis disease. Biomedicines. (2024) 12(6):1360. doi: 10.3390/biomedicines12061360, PMID: 38927567 PMC11202132

[48] Affourtit C Carre JE. Mitochondrial involvement in sarcopenia. Acta Physiol (Oxf). (2024) 240:e14107. doi: 10.1111/apha.14107, PMID: 38304924

[49] Tu W Rao S. Mechanisms underlying T cell immunosenescence: aging and cytomegalovirus infection. Front Microbiol. (2016) 7:2111. doi: 10.3389/fmicb.2016.02111, PMID: 28082969 PMC5186782

[50] Kilgour AH Firth C Harrison R Moss P Bastin ME Wardlaw JM. Seropositivity for CMV and IL-6 levels are associated with grip strength and muscle size in the elderly. Immun Ageing. (2013) 10:33. doi: 10.1186/1742-4933-10-33, PMID: 23938060 PMC3765201

