# Supplementary material for: Association between human herpesvirus 6 status and sarcopenia risk: a UK biobank cohort study with sex-specific patterns and telomere length modification
Source: Front Immunol. 2025 Sep 17;16:1623291. doi: 10.3389/fimmu.2025.1623291 (PMC12484122; doi:10.3389/fimmu.2025.1623291)
Supplement: Supplementary file 1 [file Table1.docx]

Supplementary Table 1. Follow-up cohort characteristic.

|  | level | Non-Sarcopenia  (n=336083) | Sarcopenia  (n=2729) | p |
| --- | --- | --- | --- | --- |
| age (mean (SD)) |  | 55.060 (7.632) | 59.756 (6.673) | <0.0001 |
| Sex (%) | Female | 13347 (51.06) | 344 (38.57) | <0.0001 |
|  | Male | 12791 (48.94) | 548 (61.43) |  |
| Ethnicity (%) | Asian | 377 (1.44) | 32 (3.59) | <0.0001 |
|  | Black | 89 (0.34) | 0 (0.00) |  |
|  | Mixed | 100 (0.38) | 3 (0.34) |  |
|  | White | 25572 (97.83) | 857 (96.08) |  |
| Education level (%) | college | 12140 (46.45) | 282 (31.61) | <0.0001 |
|  | high school | 12389 (47.40) | 469 (52.58) |  |
|  | less than high school | 1609 (6.16) | 141 (15.81) |  |
| TaqMan group (%) | Negative | 25791 (98.67) | 882 (98.88) | 0.0306 |
|  | DR-only positive | 11 (0.04) | 2 (0.22) |  |
|  | Typical positive | 336 (1.29) | 8 (0.90) |  |
| Alcohol status (%) | Current | 24948 (95.45) | 832 (93.27) | 0.0004 |
|  | Never | 629 (2.41) | 40 (4.48) |  |
|  | Previous | 561 (2.15) | 20 (2.24) |  |
| Smoking status (%) | No | 11139 (42.62) | 368 (41.26) | 0.4391 |
|  | Yes | 14999 (57.38) | 524 (58.74) |  |
| Diabetes (%) | No | 25477 (97.47) | 822 (92.15) | <0.0001 |
|  | Yes | 661 (2.53) | 70 (7.85) |  |
| Overall health rating (%) | Excellent | 6245 (23.89) | 103 (11.55) | <0.0001 |
|  | Fair | 3577 (13.69) | 209 (23.43) |  |
|  | Good | 15922 (60.92) | 534 (59.87) |  |
|  | Poor | 394 (1.51) | 46 (5.16) |  |
| Albumin (mean (SD)) |  | 45.403 (2.523) | 44.857 (2.551) | <0.0001 |
| CRP (mean (SD)) |  | 2.039 (3.510) | 2.968 (4.600) | <0.0001 |

##

## Supplementary Table 2. The Cox regression analysis of HHV-6 status and sarcopenia in the follow up cycle.

|  | **Model 1**  **(Unadjusted)** | | | **Model 2**  **(Demographics Adjusted)** | | | **Model 3**  **(Fully Adjusted)** | | |
| --- | --- | --- | --- | --- | --- | --- | --- | --- | --- |
| **Characteristic** | **HR**^1^ | **95% CI**^1^ | **p-value** | **HR**^1^ | **95% CI**^1^ | **p-value** | **HR**^1^ | **95% CI**^1^ | **p-value** |
| **Baseline group** | | |  |  |  |  |  |  |  |
| Negative | — | — |  | — | — |  | — | — |  |
| DR-only positive | 3.75 | 0.94, 15.0 | 0.062 | 3.60 | 0.90, 14.4 | 0.071 | 4.76 | 1.19, 19.1 | 0.028 |
| Typical positive | 0.61 | 0.30, 1.23 | 0.2 | 0.63 | 0.32, 1.27 | 0.2 | 0.63 | 0.31, 1.26 | 0.2 |
| **Baseline female group** | | |  |  |  |  |  |  |  |
| Negative | — | — |  | — | — |  | — | — |  |
| DR-only positive | 0.00 | 0.00, Inf | >0.9 | 0.00 | 0.00, Inf | >0.9 | 0.00 | 0.00, Inf | >0.9 |
| Typical positive | 0.58 | 0.19, 1.82 | 0.4 | 0.59 | 0.19, 1.84 | 0.4 | 0.63 | 0.20, 1.96 | 0.4 |
| **Baseline male group** | | |  |  |  |  |  |  |  |
| Negative | — | — |  | — | — |  | — | — |  |
| DR-only positive | 6.41 | 1.60, 25.7 | 0.009 | 5.49 | 1.37, 22.0 | 0.016 | 7.24 | 1.80, 29.1 | 0.005 |
| Typical positive | 0.63 | 0.26, 1.53 | 0.3 | 0.66 | 0.27, 1.59 | 0.4 | 0.65 | 0.27, 1.56 | 0.3 |
| ^1^ HR = Hazard Ratio, CI = Confidence Interval | | | | | | | | | |

## Supplementary Table 3. Sensitivity analysis of the correlation between HHV-6 status and sarcopenia in the follow up cycle.

|  | **Model 1**  **(Unadjusted)** | | | **Model 2**  **(Demographics Adjusted)** | | | **Model 3**  **(Fully Adjusted)** | | |
| --- | --- | --- | --- | --- | --- | --- | --- | --- | --- |
| **Characteristic** | **OR**^1^ | **95% CI**^1^ | **p-value** | **OR**^1^ | **95% CI**^1^ | **p-value** | **OR**^1^ | **95% CI**^1^ | **p-value** |
| **Baseline group** | | |  |  |  |  |  |  |  |
| Negative | — | — |  | — | — |  | — | — |  |
| DR-only positive | 2.19 | 0.71, 6.80 | 0.2 | 2.29 | 0.74, 7.10 | 0.2 | 2.55 | 0.82, 7.93 | 0.11 |
| Typical positive | 0.97 | 0.65, 1.45 | 0.9 | 0.97 | 0.65, 1.44 | 0.9 | 0.95 | 0.64, 1.43 | 0.8 |
| **Baseline female group** | | |  |  |  |  |  |  |  |
| Negative | — | — |  | — | — |  | — | — |  |
| DR-only positive | 0.00 | 0.00, Inf | >0.9 | 0.00 | 0.00, Inf | >0.9 | 0.00 | 0.00, Inf | >0.9 |
| Typical positive | 1.02 | 0.59, 1.77 | >0.9 | 0.98 | 0.57, 1.70 | >0.9 | 1.07 | 0.62, 1.85 | 0.8 |
| **Baseline male group** | | |  |  |  |  |  |  |  |
| Negative | — | — |  | — | — |  | — | — |  |
| DR-only positive | 6.06 | 1.95, 18.8 | 0.002 | 6.58 | 2.12, 20.4 | 0.001 | 6.25 | 2.00, 19.5 | 0.002 |
| Typical positive | 0.92 | 0.51, 1.66 | 0.8 | 0.94 | 0.52, 1.70 | 0.8 | 0.90 | 0.49, 1.63 | 0.7 |
| ^1^ HR = Hazard Ratio, CI = Confidence Interval Supplementary Table 4. The correlation between EBV/CMV status and sarcopenia.  \|  \| OR (95% CI) \| p-value \| \| --- \| --- \| --- \| \| **EBV** \|  \|  \| \| Negative \| - \| - \| \| Positive \| 0.54(0.19-2.29) \| 0.3 \| \| **CMV** \|  \|  \| \| Negative \| - \| - \| \| Positive \| 1.49(0.69-3.47) \| 0.3 \| | | | | | | | | | |


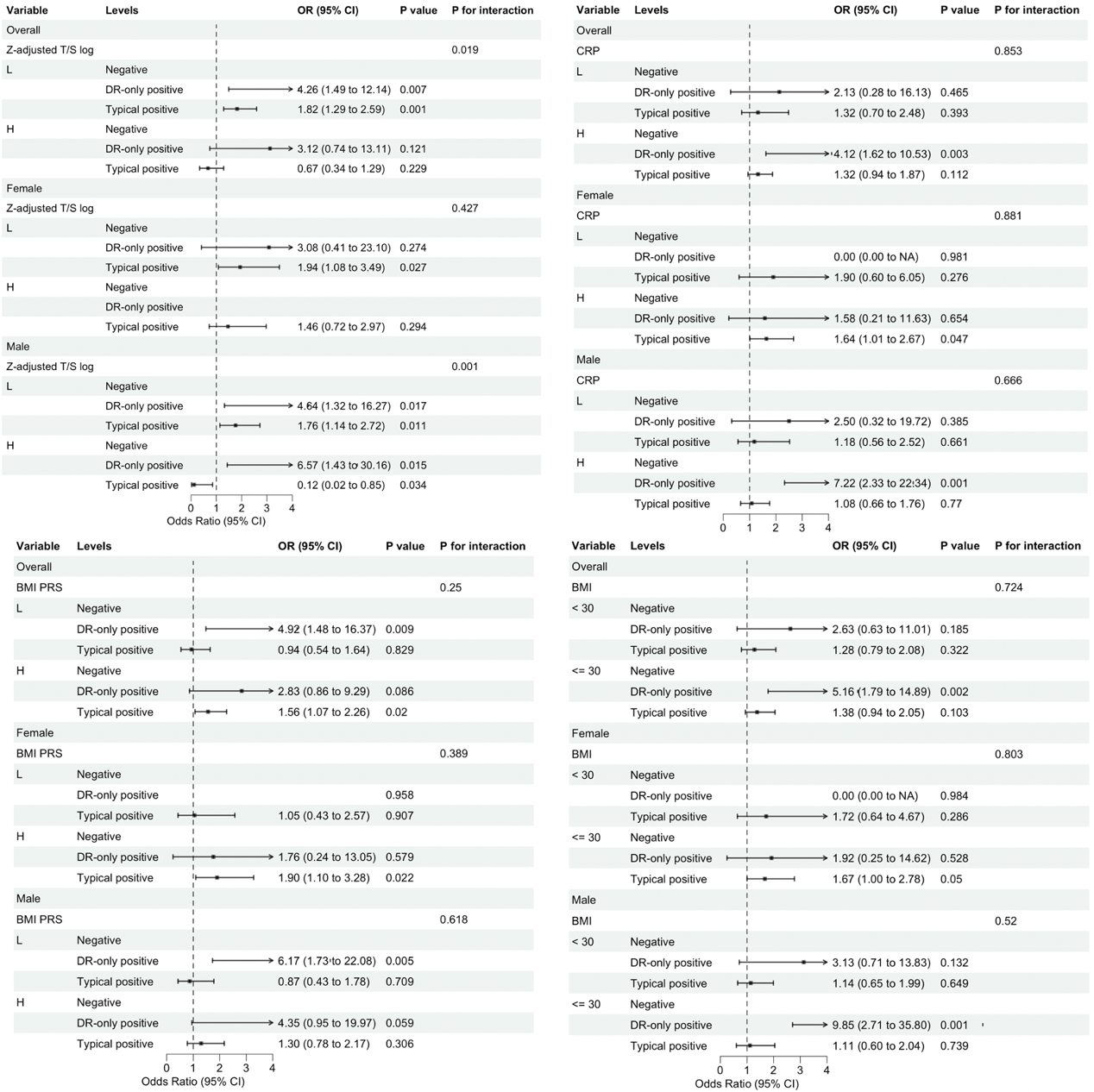


Supplementary Figure S1. Forest plot of the interaction analysis.
